# Hard Fusion Based Spectrum Sensing over Mobile Fading Channels in Cognitive Vehicular Networks

**DOI:** 10.3390/s18020475

**Published:** 2018-02-06

**Authors:** Xiaomin Qian, Li Hao, Dadong Ni, Quang Thanh Tran

**Affiliations:** 1 Key Lab of Information Coding and Transmission, Southwest Jiaotong University, Chengdu 610031, China; qxflying2009@163.com (X.Q.); dadongni@hotmail.com (D.N.); thanhktvt@gmail.com (Q.T.T.); 2 Faculty of Electrical and Electronic Engineering, University of Transport and Communications, Hanoi 117262, Vietnam

**Keywords:** cognitive radio, cognitive vehicular networks, spectrum sensing, sensing/reporting channel, correlated rayleigh fading channel, hard fusion

## Abstract

An explosive growth in vehicular wireless applications gives rise to spectrum resource starvation. Cognitive radio has been used in vehicular networks to mitigate the impending spectrum starvation problem by allowing vehicles to fully exploit spectrum opportunities unoccupied by licensed users. Efficient and effective detection of licensed user is a critical issue to realize cognitive radio applications. However, spectrum sensing in vehicular environments is a very challenging task due to vehicle mobility. For instance, vehicle mobility has a large effect on the wireless channel, thereby impacting the detection performance of spectrum sensing. Thus, gargantuan efforts have been made in order to analyze the fading properties of mobile radio channel in vehicular environments. Indeed, numerous studies have demonstrated that the wireless channel in vehicular environments can be characterized by a temporally correlated Rayleigh fading. In this paper, we focus on energy detection for spectrum sensing and a counting rule for cooperative sensing based on Neyman-Pearson criteria. Further, we go into the effect of the sensing and reporting channel conditions on the sensing performance under the temporally correlated Rayleigh channel. For local and cooperative sensing, we derive some alternative expressions for the average probability of misdetection. The pertinent numerical and simulating results are provided to further validate our theoretical analyses under a variety of scenarios.

## 1. Introduction

Vehicular network (VN) serves as an actual enabling application that is conceived to enhance road safety and provide in-vehicle infotainment by allowing vehicle-to-vehicle (V2V) as well as vehicle-to-infrastructure (V2I) communications. It has attracted considerable investigation during the past few years but still faces a host of challenges before starting the actual implementation. One of the major challenges is the deficient frequency resources available for wireless communications in vehicular networks.

Currently, the Federal Communication Commission (FCC) has reserved 75-MHz licensed spectrum bandwidth (i.e., seven 10-MHz channels) at a center frequency of 5.9-GHz in support of dedicated short-range communication (DSRC) in vehicular environments. However, a tremendous increase in wireless applications being developed for vehicular communications, may give rise to serious congestion of the band, and ultimately reducing the communication efficient for safety applications. Moreover, not only road safety applications, but also the increasing number of applications related to infotainment systems will also lead to channel contention and spectrum deficiency. In view of the stringent QoS requirements on DSRC spectrum, it is not sufficient for all applications to depend only on the 5.9-GHz DSRC spectrum. There is a dramatic increase in the demand for frequency resources to satisfy their communication requirements.

To solve the aforementioned problems, cognitive radio (CR) [1,2] has increasingly been presented as a potential technique being capable of accessing licensed but unoccupied frequency bands only without causing any unacceptable interference to licensed users (or primary users, PUs). Cognitive radio has become one of the most breathtaking technologies to improve the spectrum efficiency effectively for the past couple of decades. In addition to a better remedy for frequency scarcity issue, cognitive radio is appropriate for vehicular environments, since their unique characteristics make it much better to achieve the spatial and temporal reusage of the empty frequency bands of PUs compared to other traditional cognitive networks [3,4]. To efficiently reutilize the spectrum holes with minimum interference to PUs, the CR-enabled vehicles, which can be called secondary vehicular users (SVUs), need to reliably make a decision inferring the presence or absence of the PU. Consequently, spectrum sensing constitutes the key component of cognitive vehicular networks (CVNs) [5].

Spectrum sensing performed in temporal domain [6] and spatial domain [7,8] is a wealthily investigated subject. Among them, cooperative sensing [9] has already attracted strong research interest as it is such an effective way to improve sensing accuracy and efficiency by exploiting multi-user cooperative spatial diversity. However, these existing sensing techniques are largely concentrated in the traditional CR networks. They assume that all users just stand in one place. As a result, they cannot be directly applicable to vehicular networks. In CVNs, we must give an account of the idiosyncrasies of vehicular networks such as high mobility while designing sensing schemes [3,10]. For instance, rapid movement of vehicles makes the availability of spectrum holes dynamically change since a vehicle may enter or leave a region interfered by a particular PU at different locations along the road. In this regard, it is considerable for SVUs to detect PU activities in the fastest possible way. Additionally, the vehicles’ motion are restricted and predictable due to the fixed road topology. In consequence, each vehicle may be glad to know in advance the spectrum opportunities to better utilize them for transmission on its track. Further, high speeds and the environmental clutter can affect the received signal due to the Doppler effect, fading and shadowing. These factors will have immediate impacts on spectrum sensing of CVNs.

Spectrum sensing and sharing in dynamic environments have been researched in some preliminary works [4,11,12,13,14,15,16,17,18]. The authors in [4] proposed a novel adaptive sensing coordinated mechanism, in which the central nodes merely assist and coordinate the SVUs to better acquire the availability of spectrum holes instead of completely controlling the sensing and access. The authors in [11] studied the detection performance of spectrum sensing under the shadowing and multi-path composite fading channel in vehicular environments. The authors in [12] considered a cognitive inter-vehicular cooperative network where all channels are modelled by the double Rayleigh fading distribution. The outage probabilities in cooperative spectrum sharing networks were investigated under non-identical Rayleigh Fading channels [13,14] and Nakagami-m fading channels [15]. The authors in [16] presented an asynchronous collaborative sensing framework in which the tagged vehicle collects energy measurements labeled with time and location information from collaborative SVUs and assigns weights based on their storing time and location. The authors in [17] proposed a distributed collaborative sensing scheme based on adaptive decision threshold for sensing and voting scheme for connected vehicles. An integrated overview of spectrum sensing in cognitive vehicular environments can be discovered in [18]. None of this previous research considered the effect of temporal correlation due to vehicle motion and multi-path propagation in a mobile vehicular environment. Moreover, the reporting channels between SVUs and the fusion center (FC) were assumed to be ideal.

In this paper, our attention is centered on the large-scale fixed PU detection in an infrastructure-based CVN. Each SVU periodically performs sensing and reports its sensing information via the dedicated reporting channel to the nearby FC. The FC fuses the received information to make a global decision for the current cell. At this moment, some vehicles taking part in cooperative sensing may have left this cell. Based on mutual benefit, we allow the back vehicles to utilize the spectrum availability information after the front vehicles sensed. We investigate the sensing performance using hard fusion [19]. Although soft fusion can gather improved performance more than hard fusion, the burden of reporting overhead impedes its applicability [20]. The main contribution of our work is the following:

1. investigate the effect of fading correlation on spectrum sensing performance over temporally correlated Rayleigh sensing channel;
2. make clear how dramatically the reporting channel conditions could influence the reliability of a local/global decision, when made by the FC.

For local and cooperative sensing, we evaluate the sensing performances by means of theoretical calculations or Monte Carlo simulations. Our results show that poor channel condition harms the detection performance. On the other hand, we will demonstrate that, if utilized properly, the fast time-varying fading caused by the Doppler spread can be used to enhance the detection performance by taking advantage of temporal diversity.

The rest of the paper is organized as follows. In Section 2, we build up the system model and make some of the assumptions about spectrum sensing in vehicular environments. In Section 3, the local sensing performance over correlated Rayleigh fading is analyzed. In Section 4, we consider the cooperative sensing performance with the fading reporting channels. This is followed by the numerical and simulated results in Section 5. Lastly, we summarize the conclusions in Section 6.

## 2. System Model and Problem Formulation

In this section, we briefly introduce the network model, the channel model, the sensing model, and the assumptions made in the CVNs under consideration.

### 2.1. Network Model

**Figure 1:**
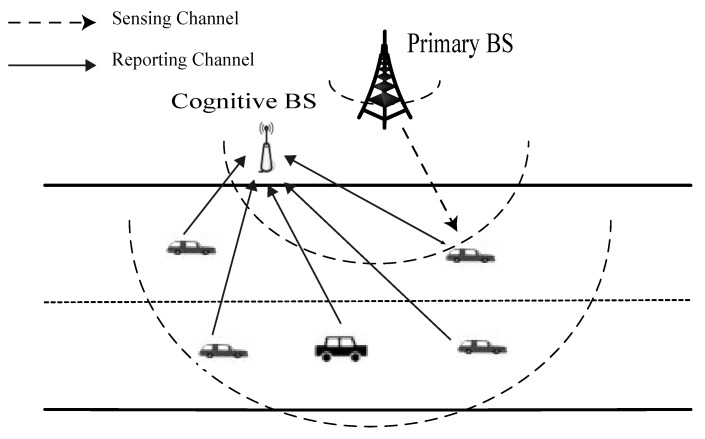
An illustrative example of cooperative sensing operation for cognitive vehicular networks. There is a PU, a cognitive BS and multiple cooperative SVUs over wireless sensing/reporting channels.

We consider a vehicular network in multi-lane highway scenarios in which licensed and cognitive users coexisting peacefully within the same geospatial region, like the one depicted in Figure 1. We consider a large-scale fixed licensed user case where the PU accesses channel with probability *p*. The cognitive network is an infrastructure-aided network, in which each cell is composed of a cognitive base station (or fusion center) and a number of associated SVUs. The FC coordinates SVUs collaborative sensing and their access to a vacant PU channel. Each SVU periodically performs sensing and reports its sensing information via the dedicated reporting channel to the nearby FC. According to the received local sensing results, the FC comes up with a global decision for the current cell. At this moment, some vehicles engaged in cooperative sensing may have left this cell. Hence, the FC diffuses the global decision to the next passing SVUs. We assume that the SVUs are Poisson distributed and follow the freeway mobility model. The vehicles move independently from any other, and thus, the sensing channels between PU and SVUs are all independent of one another.

### 2.2. Channel Model

With the far-field assumption, the distances between the PU and SVUs are wide apart in comparison with the range of CVN. As a result, we can assume that the distances from the PU to the SVUs are approximately identical. We will consider only the small-scale fading.

A mobile Rayleigh fading channel is usually used to characterize the channel time variations. We can model the channel vector as a zero-mean, unit-variance, complex Gaussian random vector with the land-mobile correlation fading model (Table 2.1 in [21]). Let ρ(τ) denote the correlation coefficient between two samples separating τ time interval, which satisfies

(1) $$\rho \left(\tau \right)=J_{0}\left(2\pi F_{d}T_{s}\tau \right),$$

where $J_{v}\left(\cdot \right)$ is defined by the *v*th-order Bessel function of the first kind, and $F_{d}T_{s}$ represents the normalized Doppler shift, $F_{d}T_{s}=\frac{f_{c}v}{c}T_{s}$, which may relate to a vehicle speed *v*, a carrier frequency $f_{c}$ and the sampling interval $T_{s}$. Note that ρ = 1 means a time-invariant channel, ρ = 0 means a completely random time-variant channel.

In general, the channel correlation characteristic is mainly dependent on $F_{d}T_{s}$. Based on the different $F_{d}T_{s}$, we can employ a suitable model, which is convenient to analyze the fading characteristic of sensing channel. For instance, three different models, $M_{1}$, $M_{2}$ and $M_{3}$, are given as follows, respectively

$M_{1}$: When $F_{d}T_{s}$ is relatively smaller (<0.001), the channel process is nearly time-invariant.

$M_{2}$: When $F_{d}T_{s}$ is small (<0.03), the channel process is correlated (“slow” fading).

$M_{3}$: For larger values of $F_{d}T_{s}$ (>0.03), the channel process are almost independent time-varying (“fast” fading).

It should be pointed out that $M_{1}$ and $M_{3}$ are two extreme cases of temporally correlated Rayleigh fading channel.

### 2.3. Sensing Model

Spectrum sensing is a binary hypothesis detection issue, with the null ($H_{0}$) and alternative ($H_{0}$) hypotheses associated to the absence and presence of PU in a given frequency band, respectively. We consider a cognitive vehicular network consisting of *N* collaborative SVUs. Assume *L* sampling observations can be used within a sensing interval. Under both hypotheses, the received observations by the *i*-th SVU can then be expressed as, respectively

(2) $$\begin{array}{c} x_{i}\left(k\right)=\left\{\begin{array}{cc} n_{i}\left(k\right) & H_{0}\\ h_{i}\left(k\right)s\left(k\right)+n_{i}\left(k\right) & H_{1} \end{array}\right., \end{array}$$

where s(k) is the signal from the PU, $x_{i}\left(k\right)$ is the received signal by the *i*-th SVU, $h_{i}\left(k\right)$ is the channel gain between the PU and the *i*-th SVU with $E[{\left|h_{i}\left(k\right)\right|}^{2}]=1$, and $n_{i}\left(k\right)$ is the complex additive white Gaussian noise (AWGN) with mean zero and variance $\sigma_{n}^{2}$, i.e., $n_{i}\left(k\right)∼CN\left(0,\sigma_{n}^{2}\right)$. And, s(k), $h_{i}\left(k\right)$ and $n_{i}\left(k\right)$ are assumed to be independent of each other, which is reasonable for a practical situation.

To reduce the complexity and reporting channel overhead, each SVU employs a mapping rule to its observations, to produce a quantized information denoted by $q(x_{i})$. In this paper, we suppose each SVU makes a binary decision $u_{i}=q\left(x_{i}\right)\in \left\{+1,-1\right\}$ with probabilities of false-alarm and mis-detection $P_{fi}$ and $P_{mi}$. These decisions are then reported to the FC via a fading reporting channel or link, bit error may happen, which further affects the sensing performance at the FC. We will model the reporting channel as a Rayleigh fading channel [22]. The received observation at the FC from the *i*-th SVU can be described as

(3) $$\begin{array}{c} z_{i}=g_{i}u_{i}+w_{i}, \end{array}$$

where $g_{i}$ is the fading gain of reporting channel and $w_{i}$ is a zero-mean Gaussian random variable with variance $\delta_{i}^{2}$, i.e., $w_{i}∼N\left(0,\delta_{i}^{2}\right)$. Once the noisy observation $\left\{z_{i};i=1,2,\cdots ,N\right\}$ is received and decoded, the FC makes a global decision on which hypothesis is more likely to be true.

In the ordinary sense, an optimal fusion rule for hard combination behaves in the form of a counting rule [23]. This argument proves to be true even when those decisions are received via unreliable communication channels, provided that the channels are also independent and identical distribution (IID). Later in this paper, we focus on energy detection for spectrum sensing and look at the detection performance of the counting rule in Rayleigh fading channels.

## 3. Local Sensing with Energy Detection

**Figure 2:**
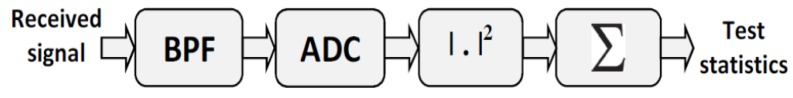
Digital energy detector model.

The spectrum sensing techniques often employed for local sensing are energy detection, cyclostationary detection and matched filter. The matched filter (also referred to as coherent detector) is widely regarded as the optimum approach but it relies heavily on the accurate *priori* knowledge about the PU signal, which is hard to be obtained. Cyclostationary detection can distinguish the PU signal from noise at very low signal-to-noise ratio (SNR) but still needs some *priori* knowledge about the PU signal. Energy detection [24,25] is the most practical method because it merely estimates the signal energy on the considered band and produces good performance without any *priori* information about the PU signal, as illustrated in Figure 2. Furthermore, energy detection is a viable choice for vehicular networks on account of its high mobile environment and low latency tolerance.

When digital energy detector is adopted, the corresponding energy statistic at the *i*-th SVU, denoted by $e_{i}$, is expressed as

(4) $$\begin{array}{c} e_{i}=\sum_{k=0}^{L-1}|x_{i}{\left(k\right)|}^{2}. \end{array}$$

where *L* corresponds to the number of samples within a sensing interval.

Let λ denote the decision threshold for energy detector and $u_{i}$ denote the local decision, the decision rule at each SVU is represented as

(5) $$\begin{array}{c} u_{i}=\left\{\begin{array}{cc} +1\; & if\;e_{i}\geq \lambda \\ -1\; & if\;e_{i}<\lambda \end{array}\right.. \end{array}$$

This means that when $e_{i}\geq \lambda$, SVU *i* makes its individual decision $u_{i}$ = +1 which tells the PU signal is detected ($H_{1}$); otherwise, it makes a decision $u_{i}$ = −1 which declares that the PU signal is not detected ($H_{0}$).

When *L* is sufficiently large, based on the Central Limit Theorem (CLT), the energy statistic $e_{i}$ in (4) can be described by a Gaussian distribution under both hypotheses $H_{0}$ and $H_{1}$ [24]. Let $E_{s}\;=\sum_{k=0}^{L-1}{\left|s(k)\right|}^{2}$ denote the transmitted signal energy by the PU. The corresponding mean and variance are given by

(6) $$\begin{array}{c} \left\{\begin{array}{ccc} \begin{array}{c} H_{0}\\ H_{1} \end{array} & \begin{array}{c} \mu_{0}=L\sigma_{n}^{2}\\ \mu_{1}=\left(L+\eta_{i}\right)\sigma_{n}^{2} \end{array} & \begin{array}{c} \sigma_{0}^{2}=L\sigma_{n}^{4}\\ \sigma_{1}^{2}=\left(L+2\eta_{i}\right)\sigma_{n}^{4} \end{array} \end{array}\right., \end{array}$$

where

(7) $$\eta_{i}=\frac{1}{L}\sum_{k=0}^{L-1}{\left|h_{i}\left(k\right)\right|}^{2}\frac{E_{s}}{\sigma_{n}^{2}}=\frac{1}{L}\sum_{k=0}^{L-1}{\left|h_{i}\left(k\right)\right|}^{2}\eta_{s},$$

In the above equation, the term $\eta_{i}$ denotes the instantaneous SNR experienced by the *i*-th SVU. It is straightforward to see that $\eta_{i}$ is very different from (sensing) period to period, since it relies heavily on the fading characteristics of the wireless channel. In addition, the term $\eta_{s}=\frac{E_{s}}{\sigma_{n}^{2}}$ denotes the local average SNR. To be emphasized, the SNR ($\eta_{s}$) is *L* times more than the average SNR measured at the energy detector output, which can be expressed as $\eta_{0}=\frac{E_{s}}{L\sigma_{n}^{2}}$ [26].

Subsequently, the probabilities of false alarm, detection and miss detection for a given threshold (λ) at the *i*-th SVU can be deduced as follows, respectively

(8) $$P_{fi}\left(\lambda \right)=P\left(e_{i}\geq \lambda |H_{0}\right)=Q\left(\frac{\lambda -L\sigma_{n}^{2}}{\sqrt{L}\sigma_{n}^{2}}\right),$$

(9) $$P_{di}\left(\lambda \right)=P\left(e_{i}\geq \lambda |H_{1}\right)=Q\left(\frac{\lambda -\left(L+\eta_{i}\right)\sigma_{n}^{2}}{\sqrt{L+2\eta_{i}}\sigma_{n}^{2}}\right),$$

(10) $$P_{mi}\left(\lambda \right)=1-P_{di}\left(\lambda \right)=Q\left(\frac{\left(L+\eta_{i}\right)\sigma_{n}^{2}-\lambda }{\sqrt{L+2\eta_{i}}\sigma_{n}^{2}}\right),$$

where Q(x) stands for the standard Q-function, i.e., $Q\left(x\right)=\frac{1}{\sqrt{2\pi }}\int_{x}^{\infty }\exp(-\frac{t^{2}}{2})dt$.

It is worthwhile mentioning that, $P_{fi}$ in (8) is independent of received SNR because $P_{fi}$ is considered for the hypothesis of no PU signal transmission. On the other hand, $P_{mi}$ in (10) is a conditional probability depending on instantaneous received SNR $\eta_{i}$. In this circumstance, the average $P_{m}$ can be calculated by integrating the conditional $P_{mi}$ in the AWGN case over the SNR fading distribution [24].

From (8) and (10), we also see that, λ can be analytically set to maintain the desired $P_{f}$ if $P_{f}$ is designated as the constraint of the detection problem. Then, we can obtain $P_{mi}$ related to the desired $P_{f}$ as follows:

(11) $$\begin{array}{cc} P_{mi}\left(\eta_{i}\right) & =Q\left(\frac{\eta_{i}-\sqrt{L}Q^{-1}\left(P_{f}\right)}{\sqrt{L+2\eta_{i}}}\right), \end{array}$$

where $Q^{-1}\left(x\right)$ stands for the inverse of the Gaussian Q-function.

In the following, let us begin by considering the case of $M_{1}$ where the channel gain $h_{i}\left(k\right)$ is time-invariant during the sensing interval, i.e., $h_{i}\left(k\right)=h_{i}$ for k=1,2,⋯,L. Then, $\eta_{i}$ in (7) can be rewritten as

(12) $$\begin{array}{c} \eta_{i}\;=\;{\left|h_{i}\right|}^{2}\frac{E_{s}}{\sigma_{n}^{2}}\;=\;{\left|h_{i}\right|}^{2}\eta_{s}. \end{array}$$

Under Rayleigh fading, the received instantaneous SNR $\eta_{i}$ can be viewed as exponentially distributed

(13) fηi(ηi)=1ηse−ηiηηiηs.

Under the assumptions of $E[{\left|h_{i}\right|}^{2}]=1$, for the convenience of computing, we can make the following approximation for Equation (11) as

(14) $$\begin{array}{cc} P_{mi}\left(\eta_{i}\right) & \approx Q\left(\frac{\eta_{i}-\sqrt{L}Q^{-1}\left(P_{f}\right)}{\sqrt{L+2\eta_{s}}}\right). \end{array}$$

For brevity of the presentation, in the following sections we will define

$$A=\frac{1}{\sqrt{L+2\eta_{s}}},B=\frac{\sqrt{L}Q^{-1}\left(P_{f}\right)}{\sqrt{L+2\eta_{s}}},$$

then

(15) $$P_{mi}\left(\eta_{i}\right)\approx Q\left(A\eta_{i}-B\right).$$

The average $P_{m}$ in the case of $M_{1}$, ${\bar{P}}_{m}$, can be evaluated by averaging (15) over (13) with the change of variable x=Aη−B. Consequently, this yields

(16) P¯m=∫0∞Pm(ηi)fηi(ηi)dηi≈1ηs∫0∞QAηi−Be−ηiηηsηsdηi=1Aηs∫−B∞Qxe−x+BAηsdx.

With the aid of the integrating properties of Q-function [27], and after some mathematical manipulations, we can deduce the approximate closed-form expression for ${\bar{P}}_{m}$,

(17) $$\begin{array}{c} {\bar{P}}_{m}\approx Q\left(-B\right)-e^{-\frac{B}{A\eta_{s}}}e^{\frac{1}{2A^{2}\eta_{s}^{2}}}Q\left(\frac{1}{A\eta_{s}}-B\right). \end{array}$$

In the case of $M_{2}$, in order to obtain the analytic expression for ${\bar{P}}_{m}$ based on using the PDF approach, it is desirable to obtain the PDF of $\eta_{i}$ given in (7). However, to the best of our knowledge, there is no analytic expression available for such a distribution. Therefore, it makes things computationally very difficult to find an analytic expression for ${\bar{P}}_{m}$ with the existence of time correlation. Instead of deriving an analytic expression for ${\bar{P}}_{m}$, we estimate ${\bar{P}}_{m}$ by means of Monte Carlo simulations.

In the case of $M_{3}$, the sampling observations of the fading channel $\left\{h_{i}\left(k\right)\right\}$ are completely independent of each other. $h_{i}\left(k\right)$ can be modeled as a complex Gaussian random variable with mean zero and variance $\sigma_{h}^{2}=1$, i.e., $h_{i}\left(k\right)∼CN\left(0,\sigma_{h}^{2}\right)$. Thus, $\eta_{i}$ given by (7) can be reduced to the following equation

(18) $$\eta_{i}=\frac{\sigma_{h}^{2}E_{s}}{\sigma_{n}^{2}}=\sigma_{h}^{2}\eta_{s}.$$

It is plain to see that $\eta_{i}$ is independent of instantaneous fading statistics. Thus, the ${\bar{P}}_{m}$ in the case of $M_{3}$ can be computed directly by (11) and (18).

## 4. Cooperative Sensing with Counting Rule

The concept of cooperative spectrum sensing is to utilize multiple SVUs at different locations and fuse their independent sensing messages into one unified decision about the existence of the PU. In this section, we ponder this approach based on hard fusion, and also look at the impact of the reporting channel fading to the global detection performance.

### 4.1. Equivalent Local Probability of False Alarm and Misdetection

In consideration of the unreliable characteristic of the reporting channels, let us determine its effect on the reliability of the local decision made by the FC. Let $v_{i}$ denote the decoded version of $z_{i}$ for the *i*-th SVU at the FC, the decoded rule can be expressed as

(19) $$\begin{array}{c} v_{i}=\left\{\begin{array}{cc} 1\; & if\;z_{i}\geq 0\\ 0\; & if\;z_{i}<0 \end{array}\right.. \end{array}$$

And, more remarkable, under the hypothesis $H_{j}\left(j=0,1\right)$

(20) $$\begin{array}{cc} E[v_{i}\left|H_{j}\right.] & =P\left(v_{i}=1\left|H_{j}\right.\right)\times 1+P\left(v_{i}=0\left|H_{j}\right.\right)\times 0\\ & =P(z_{i}\geq 0\left|H_{j}\right.), \end{array}$$

(21) $$\begin{array}{cc} D[v_{i}\left|H_{j}\right.] & =E\left[v_{i}^{2}\left|H_{j}\right.\right]-E{\left[v_{i}\left|H_{j}\right.\right]}^{2}\\ & =P\left(z_{i}\geq 0\left|H_{j}\right.\right)-{\left(P(z_{i}\geq 0\left|H_{j}\right.)\right)}^{2}, \end{array}$$

where E[x], D[x] denotes the expectation and variance operator with respect to *x*, respectively.

As can be seen from (20) and (21), the expectation and variance of the received decision $v_{i}$ are directly dependent on the probability $P(z_{i}\geq 0\left|H_{j}\right.)$, which can also be referred to the equivalent probabilities of false-alarm and misdetection of local sensing. For notational convenience, let us denote these probabilities as $P_{Fi}$ and $P_{Di}$ under both hypotheses. In order to capture the statistical properties of $v_{i}$, we first need to obtain the probabilities $P_{Fi}=f\left(z_{i}\geq 0|H_{0}\right)$ and $P_{Di}=f\left(z_{i}\geq 0|H_{1}\right)$.

Clearly, under the hypothesis $H_{0}\left(H_{1}\right)$, $u_{i}$ obeys the following distribution with the parameter of $P_{fi}\left(P_{di}\right)$.

$$P\left(u_{i}\left|H_{0}\right.\right)=\left\{\begin{array}{cc} P_{fi} & u_{i}=+1\\ 1-P_{fi} & u_{i}=-1 \end{array}\right.,$$

$$P\left(u_{i}\left|H_{1}\right.\right)=\left\{\begin{array}{cc} P_{di} & u_{i}=+1\\ 1-P_{di} & u_{i}=-1 \end{array}\right..$$

Then, under hypotheses $H_{0}$ and $H_{1}$, the equivalent probability of false alarm and misdetection can be written as:

(22) $$\begin{array}{cc} P_{Fi}=f\left(z_{i}\geq 0\left|H_{0}\right.\right) & =\underset{u_{i}\in \left\{+1,-1\right\}}{\Sigma }f\left(z_{i}\geq 0\left|u_{i}\right.\right)P\left(u_{i}\left|H_{0}\right.\right)\\ & =f\left(z_{i}\geq 0\left|u_{i}=+1\right.\right)P_{fi}+f\left(z_{i}\geq 0\left|u_{i}=-1\right.\right)\left(1-P_{fi}\right), \end{array}$$

(23) $$\begin{array}{cc} P_{Di}=f\left(z_{i}\geq 0\left|H_{1}\right.\right) & =\underset{u_{i}\in \left\{+1,-1\right\}}{\Sigma }f\left(z_{i}\geq 0\left|u_{i}\right.\right)P\left(u_{i}\left|H_{1}\right.\right)\\ & =f\left(z_{i}\geq 0\left|u_{i}=+1\right.\right)P_{di}+f\left(z_{i}\geq 0\left|u_{i}=-1\right.\right)\left(1-P_{di}\right). \end{array}$$

Without loss of generality, suppose that the reporting channels between SVUs and FC is also Rayleigh fading channel draw from CSCG distribution CN(0,2). In other words, the PDF of the channel gain $g_{i}$ can be represented as

(24) $$f\left(g_{i}\right)=\left\{\begin{array}{cc} g_{i}\exp\left(-\frac{g_{i}^{2}}{2}\right) & g_{i}\geq 0\\ 0 & g_{i}<0 \end{array}.\right.$$

On the basis of the fact that $y_{i}=g_{i}u_{i}$, $z_{i}=y_{i}+w_{i}$ and $w_{i}$ is a Gaussian random variable with mean zero and variance $\delta_{i}^{2}$, we can easily get

(25) $$f\left(z_{i}\left|y_{i}\right.\right)=\frac{1}{\sqrt{2\pi }\delta_{i}}\exp\left(-\frac{{\left(z_{i}-y_{i}\right)}^{2}}{2\delta_{i}^{2}}\right),$$

(26) $$f\left(y_{i}\left|u_{i}\right.\right)=u_{i}y_{i}\exp\left(-\frac{y_{i}^{2}}{2}\right)I\left(u_{i}y_{i}\right),$$

where I(·) denotes the indicator function given by

$$I\left(t\right)=\left\{\begin{array}{cc} 1\; & if\;t\geq 0\\ 0\; & if\;t<0 \end{array}.\right.$$

Further still, according to this fact that $f\left(z_{i}\left|y_{i},u_{i}\right.\right)=f\left(z_{i}\left|y_{i}\right.\right)$, which stemmed from the fact that $\left(g_{i},u_{i}\right)\to y_{i}\to z_{i}$ is a Markov chain, we can safely come to the following result

(27) $$\begin{array}{cc} f(z_{i}\left|u_{i}\right.) & =\frac{f(z_{i},u_{i})}{p(u_{i})}=\frac{\int f\left(z_{i}\left|y_{i},u_{i}\right.\right)f\left(y_{i}\left|u_{i}\right.\right)p\left(u_{i}\right)dy_{i}}{p(u_{i})}\\ & =\int f\left(z_{i}\left|y_{i}\right.\right)f\left(y_{i}\left|u_{i}\right.\right)dy_{i}. \end{array}$$

Then, substituting (25) and (26) into (27), we can obtain the conditional PDF of the received observation $z_{i}$, given the local decision $u_{i}=+1$, as

(28) $$\begin{array}{cc} f(z_{i}\left|u_{i}=+1\right.) & =\int_{0}^{\infty }\frac{1}{\sqrt{2\pi }\delta_{i}}\exp\left(-\frac{{\left(z_{i}-y_{i}\right)}^{2}}{2\delta_{i}^{2}}\right)y_{i}\exp\left(-\frac{y_{i}^{2}}{2}\right)dy_{i}\\ & =\frac{1}{\sqrt{2\pi }\delta_{i}}\exp\left(-\frac{{z_{i}}^{2}}{2(1+\delta_{i}^{2})}\right)\int_{0}^{\infty }y_{i}\exp\left(-\frac{{\left(y_{i}-\frac{z_{i}}{1+\delta_{i}^{2}}\right)}^{2}}{\frac{2\delta_{i}^{2}}{1+\delta_{i}^{2}}}\right)dy_{i}. \end{array}$$

More specifically, performing a change of variable $t=y_{i}-\frac{z_{i}}{1+\delta_{i}^{2}}$ and making the best of the integral properties of Q-function, we can obtain

(29) $$\begin{array}{c} f\left(z_{i}\left|u_{i}=+1\right.\right)=\frac{C^{2}\delta_{i}^{3}}{\sqrt{2\pi }}\exp\left(-\frac{z_{i}^{2}}{2\delta_{i}^{2}}\right)\left[1+\sqrt{2\pi }Cz_{i}\exp\left(\frac{C^{2}z_{i}^{2}}{2}\right)Q\left(-Cz_{i}\right)\right], \end{array}$$

where $C=\frac{1}{\delta_{i}\sqrt{1+\delta_{i}^{2}}}$, $Q\left(-x\right)=1-Q\left(x\right)$.

Integrating both sides of (29) with respect to $z_{i}$, we can obtain

(30) $$\begin{array}{c} P\left(z_{i}\geq 0\left|u_{i}=+1\right.\right)=\int_{0}^{\infty }f(z_{i}\left|u_{i}=+1\right.)dz_{i}\\ =\frac{C^{2}\delta_{i}^{3}}{\sqrt{2\pi }}\int_{0}^{\infty }\left[\exp\left(-\frac{z_{i}^{2}}{2\delta_{i}^{2}}\right)+\frac{\sqrt{2\pi }}{\delta_{i}\sqrt{1+\delta_{i}^{2}}}z_{i}\exp\left(-\frac{{z_{i}}^{2}}{2(1+\delta_{i}^{2})}\right)Q\left(-\frac{z_{i}}{\delta_{i}\sqrt{1+\delta_{i}^{2}}}\right)\right]dz_{i}\\ =\frac{\delta_{i}}{\sqrt{2\pi }\left(1+\delta_{i}^{2}\right)}\left[\frac{\sqrt{2\pi }\delta_{i}}{2}+\frac{\sqrt{2\pi }\sqrt{1+\delta_{i}^{2}}}{2\delta_{i}}+\frac{\sqrt{2\pi }}{2\delta_{i}}\right]\\ =\frac{1}{2}+\frac{1}{2\sqrt{1+\delta_{i}^{2}}}=\frac{1}{2}\left(1+\sqrt{\frac{\gamma_{i}}{2+\gamma_{i}}}\right), \end{array}$$

where $\gamma_{i}$ denotes the SNR of the Rayleigh fading reporting channel.

A similar analysis can be conducted for the case of $u_{i}=-1$, we have

(31) $$f\left(z_{i}\left|u_{i}=-1\right.\right)=\frac{C^{2}\delta_{i}^{3}}{\sqrt{2\pi }}\exp\left(-\frac{z_{i}^{2}}{2\delta_{i}^{2}}\right)\left[1-\sqrt{2\pi }Cz_{i}\exp\left(\frac{C^{2}z_{i}^{2}}{2}\right)Q\left(Cz_{i}\right)\right],$$

(32) $$\begin{array}{c} P\left(z_{i}\geq 0\left|u_{i}=-1\right.\right)=\int_{0}^{\infty }f(z_{i}\left|u_{i}=-1\right.)dz_{i}=\frac{1}{2}\left(1-\sqrt{\frac{\gamma_{i}}{2+\gamma_{i}}}\right). \end{array}$$

By substituting (30) and (32) in (22) and (23), and after some calculations, we can obtain the equivalent local probability of false alarm, detection and miss detection as

(33) $$\begin{array}{c} P_{Fi}=P\left(z_{i}\geq 0\left|H_{0}\right.\right)=\frac{1}{2}+\left(P_{fi}-\frac{1}{2}\right)\sqrt{\frac{\gamma_{i}}{2+\gamma_{i}}}, \end{array}$$

(34) $$\begin{array}{c} P_{Di}=P\left(z_{i}\geq 0\left|H_{1}\right.\right)=\frac{1}{2}+\left(P_{di}-\frac{1}{2}\right)\sqrt{\frac{\gamma_{i}}{2+\gamma_{i}}}, \end{array}$$

and

(35) $$\begin{array}{c} P_{Mi}=1-P_{Di}=\frac{1}{2}+\left(P_{mi}-\frac{1}{2}\right)\sqrt{\frac{\gamma_{i}}{2+\gamma_{i}}}. \end{array}$$

From (33) and (34), we can see that, if $P_{di}>\frac{1}{2}$, then $P_{Di}\;<\;P_{di}$. Only when $P_{di}<\frac{1}{2}$, then $P_{Di}\;>\;P_{di}$. In other words, the equivalent local probability of detection at the FC is high above the local probability of detection at each SVU. In the same manner, when $P_{fi}>\frac{1}{2}$, $P_{Fi}<P_{fi}$. Only when $P_{fi}<\frac{1}{2}$, the channel error "increases" $P_{Fi}$ to be higher than $P_{fi}$. Without doubt, this is achieved with an accompanied rise in the probability of detection.

Besides, we also see that, as the reporting channel becomes more unreliable (low SNR situation, i.e., SNR $\gamma_{i}$ in dB →−∞ ), the equivalent probability $P_{Di}\;\left(P_{Fi}\right)$ is close to $\frac{1}{2}$. As SNR $\gamma_{i}$ in dB →∞, $P_{Di}\;\left(P_{Fi}\right)$ comes near to $P_{di}\;\left(P_{fi}\right)$, that is to say, this is a perfect reporting channel.

### 4.2. Global Probability of False Alarm and Misdetection

Assume that the reporting channels do not interfere with each other, and the delay is negligible. Once the fusion center decodes $\left\{z_{i}=g_{i}u_{i}+w_{i};i=1,2,\cdots ,N\right\}$ and gets $\left\{v_{i};i=1,2,\cdots ,N\right\}$, a global test statistic based on the counting rule is calculated linearly as follows:

(36) $$\begin{array}{c} \Lambda =\sum_{i=1}^{N}v_{i}. \end{array}$$

And, the decision rule at the FC is represented as

(37) $$\begin{array}{c} v_{0}=\left\{\begin{array}{cc} 1\; & if\;\Lambda \geq T\\ 0\; & if\;\Lambda <T \end{array}.\right. \end{array}$$

where *T* is the global decision threshold of counting rule, which can be in the form of $T=\left\lceil \alpha N\right\rceil$ (0<α≤1). This means that *T* or more SVUs decide the hypothesis $H_{1}$, then the global decision is $H_{1}$.

From the preceding analysis, we can see that $v_{i}$ is a stochastic variable from a Bernoulli distribution with its winning probability $P_{Fi}$ ($P_{Di}$) under the hypothesis $H_{0}$ ($H_{1}$). It is noteworthy that the pairs ($P_{Fi},P_{Di}$) of different SVUs are not really the same because the pairs ($P_{fi},P_{di}$) or $\gamma_{i}$ are different. So $\left\{v_{i},i=1,2,\cdots ,N\right\}$ is a set of independent and non-identically distributed random variables. In consequence, their sum $\Lambda =\sum_{i=1}^{N}v_{i}$ may be not conformed to a Binomial distribution. This makes it difficult to derive the exact distribution of Λ. For this reason, instead of relying on the exact distribution of Λ, we exploit a computationally inexpensive gaussian approximation for the sum of independent but not identically distributed random variables, known as the Lindberg-Feller CLT [28].

**Theorem** **1.** Lindberg-Feller Central Limit Theorem (LF-CLT)

*Assume that $\left\{X_{i},i=1,2,\cdots ,N\right\}$ is a set of independently and non-identically distributed random variables with mean $E\left[X_{i}\right]=\mu_{i}$ and variance $D\left[X_{i}\right]=\delta_{i}^{2}$. Further, assume that the two following regularity conditions are satisfied*

(38) $$D\left[X_{i}\right]>\beta_{1},$$

*and*

(39) $$E\left[{\left|X_{i}-E\left[X_{i}\right]\right|}^{3}\right]<\beta_{2},$$

*where $\beta_{1}$ and $\beta_{2}$ are two positive values. Then, for sufficiently big N, $\sum_{i=1}^{N}X_{i}$ converges asymptotically to a Gaussian distribution characterized by*

(40) $$\sum_{i=1}^{N}X_{i}\to N\left(\begin{array}{cc} \sum_{i=1}^{N}\mu_{i}, & \sum_{i=1}^{N}\delta_{i}^{2} \end{array}\right).$$

For the applicability of LF-CLT, we will show how the above-mentioned Lindberg-Feller conditions are satisfied in the Appendix A. Consequently, in a large cognitive vehicular network, the LF-CLT can be used to approximately describe the distribution of the global statistic (Λ) under both hypotheses $H_{0}$ and $H_{1}$.

Because $\left\{v_{i},i=1,2,\cdots ,N\right\}$ are all independent of each other, for a large number of SVUs, *N*, on the basis of the LF-CLT [28], Λ is asymptotically Gaussian distributed with mean

(41) $$\begin{array}{c} \mu =E\left[\Lambda \right]=\sum_{i=1}^{N}E\left[v_{i}\right]=\left\{\begin{array}{cc} \sum_{i=1}^{N}P_{Fi} & H_{0}\\ \sum_{i=1}^{N}P_{Di} & H_{1} \end{array},\right. \end{array}$$

and variance

(42) $$\begin{array}{c} \sigma^{2}=D\left[\Lambda \right]=\sum_{i=1}^{N}D\left[v_{i}\right]=\left\{\begin{array}{cc} \sum_{i=1}^{N}P_{Fi}\left(1-P_{Fi}\right) & H_{0}\\ \sum_{i=1}^{N}P_{Di}\left(1-P_{Di}\right) & H_{1} \end{array}.\right. \end{array}$$

Consequently, the global probability of false alarm and misdetection at the FC can be described by

(43) $$Q_{f}=Q\left(\frac{T-\mu }{\sqrt{\sigma^{2}}}\right)=Q\left(\frac{T-\sum_{i=1}^{N}P_{Fi}}{\sqrt{\sum_{i=1}^{N}P_{Fi}\left(1-P_{Fi}\right)}}\right),$$

(44) $$Q_{m}=Q\left(\frac{\mu -T}{\sqrt{\sigma^{2}}}\right)=Q\left(\frac{\sum_{i=1}^{N}P_{Di}-T}{\sqrt{\sum_{i=1}^{N}P_{Di}\left(1-P_{Di}\right)}}\right).$$

When the prior probabilities of presence and absence of PU are equal, i.e., $P\left(H_{1}\right)=P\left(H_{0}\right)=\frac{1}{2}$, the total probability of error detection can be written as

(45) $$\begin{array}{cc} Q_{e} & =Q_{f}+Q_{m}\\ & =Q\left(\frac{T-\sum_{i=1}^{N}P_{Fi}}{\sum_{i=1}^{N}P_{Fi}\left(1-P_{Fi}\right)}\right)+Q\left(\frac{\sum_{i=1}^{N}P_{Di}-T}{\sum_{i=1}^{N}P_{Di}\left(1-P_{Di}\right)}\right). \end{array}$$

## 5. Numerical and Simulation Results

In this section, both theoretical and simulated results are provided through numerical and Monte Carlo simulations to illustrate spectrum sensing performance in cognitive vehicular networks. We run the Monte Carlo simulation results over $10^{4}$ independent trials to verify the accuracy of the developed analytical results. Note that the arbitrary parameters, *L*, *N*, $\eta_{0}$ and $\gamma_{0}$ are fit for the developed analytical results of the local (global) probability of misdetection. Therefore, for all simulation cases, we choose some simple parameters, *L*, *N*, $\eta_{0}$ and $\gamma_{0}$ to plot the local (global) probability of misdetection, and observe how they affect the system sensing performance.

Without losing generality, we consider a fixed PU case where the PU accesses channel with probability p=0.5. The PU signal is assumed to have unit power and be any kind of modulated signal with carrier frequency of 900 MHz and bandwidth of 6 MHz. The sampling frequency in energy detector is equal to the bandwidth of PU signals, at the same time, the number of sampling observations during a sensing interval is *L* = 50. We consider a Rayleigh multi-path propagation model. For all simulation cases, the channel vector can be modeled as a zero-mean, complex Gaussian random vector with correlation matrix ρ ($F_{d}T_{s}=0.01$). In addition, the average SNR of sensing/reporting channels are assumed to be the same for all SVUs, e.g., $\eta_{0}$ = −8 dB (as it is typically assumed in CVNs) and $\gamma_{0}$ = 5.5 dB (as it makes the error probability of reporting channel equal to 0.1). The performances with respect to the local (global) probability of misdetection is estimated to meet the constraint on the local (global) probability of false alarm of $P_{f}$ ($Q_{f}$) = 0.1.

**Figure 3:**
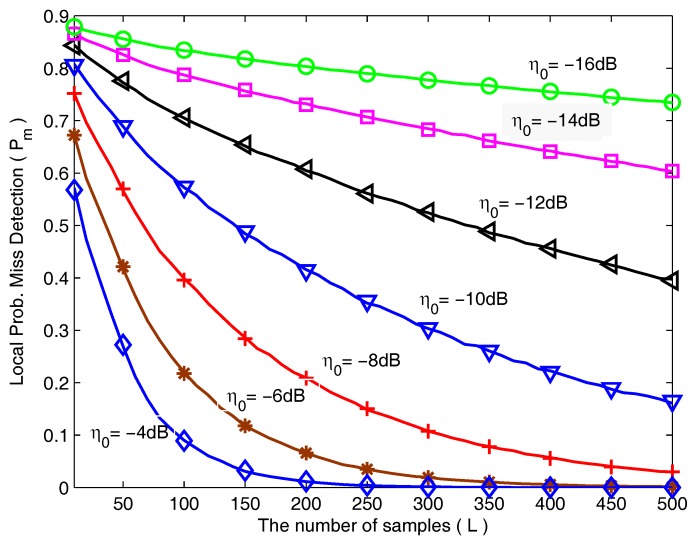
The local probability of miss detection as a function of the number of samples for various SNR.

First of all, let us consider spectrum sensing with energy detector in non-cooperative scenarios. In Figure 3, the local ${\bar{P}}_{m}$ is shown as a function of the number of samples for various SNR. The simulations are performed in the case of $M_{2}$ with the parameters $F_{d}T_{s}$ = 0.01 and $P_{f}$ = 0.1. As expected, the local ${\bar{P}}_{m}$ decreases along with the increasing number of samples. And the decrease of the local ${\bar{P}}_{m}$ in the case of high SNR is very rapid compared with low SNR.

**Figure 4:**
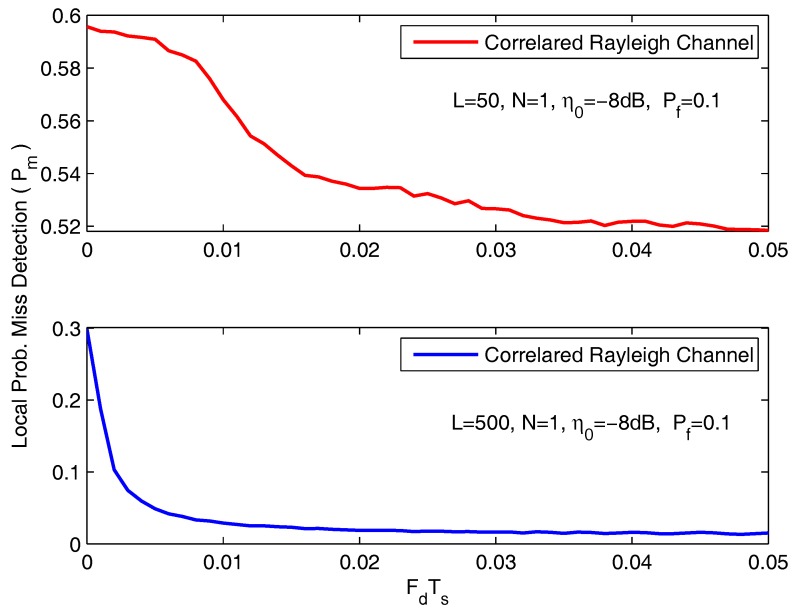
The local probability of misdetection as a function of $F_{d}T_{s}$.

In Figure 4, the local ${\bar{P}}_{m}$ is shown as a function of Doppler shift $F_{d}T_{s}$. The simulation parameters are *L* = 50/500, *N* = 1, $\eta_{0}$ = −8 dB and $P_{f}$ = 0.1. With the carrier frequency and the sampling interval fixed, $F_{d}T_{s}$ value varies at a rate linearly proportional to the speed of the SVU. In addition, a larger $F_{d}T_{s}$ results in a smaller correlation coefficient (ρ). The results show clearly that, the probability of misdetection is a decreasing function of the speed of the SVU for all the system configurations. In addition, the local ${\bar{P}}_{m}$ with 500 samples declines drastically compared with 50 samples. This reason is that high mobility and a greater number of samples give us an opportunity to achieve more received signal information, thereby enables SVUs to obtain higher sensing performance.

**Figure 5:**
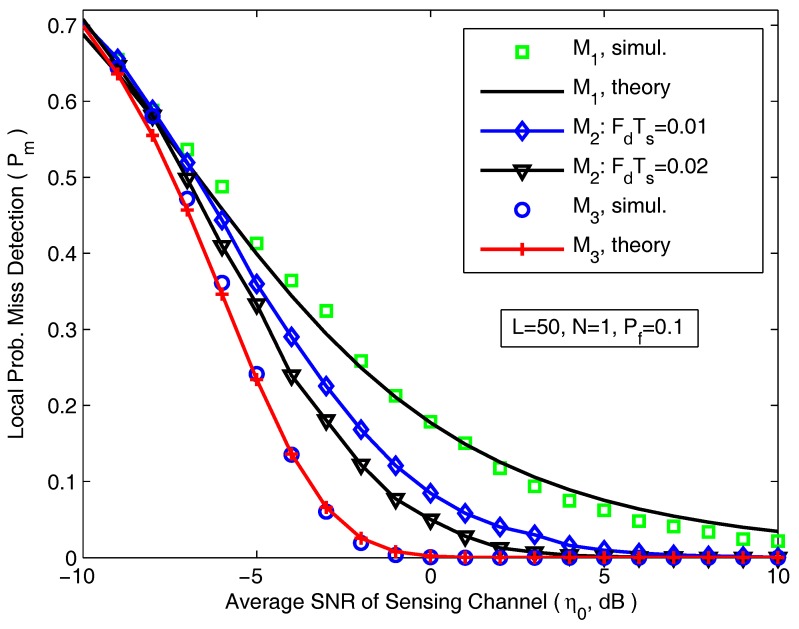
The local probability of misdetection for various average SNR of sensing channels.

In Figure 5, the local ${\bar{P}}_{m}$ is shown as a function of the average SNR of sensing channels for various values of $F_{d}T_{s}$. The simulation parameters are *L* = 50, *N* = 1 and $P_{f}$ = 0.1. The capability of energy detector decreases quickly with the reduction of the average received SNR from 10 dB to −10 dB. It can be seen that the simulating results show good or very good agreement with the theoretical analysis for both cases $M_{1}$ and $M_{3}$, confirming the validity of the developed theoretical analysis. Figure 5 also indicates that these asymptotic results (17) and (11) (18) serve as a upper and lower bounds for the local ${\bar{P}}_{m}$ for spectrum sensing over correlated Rayleigh fading channel.

**Figure 6:**
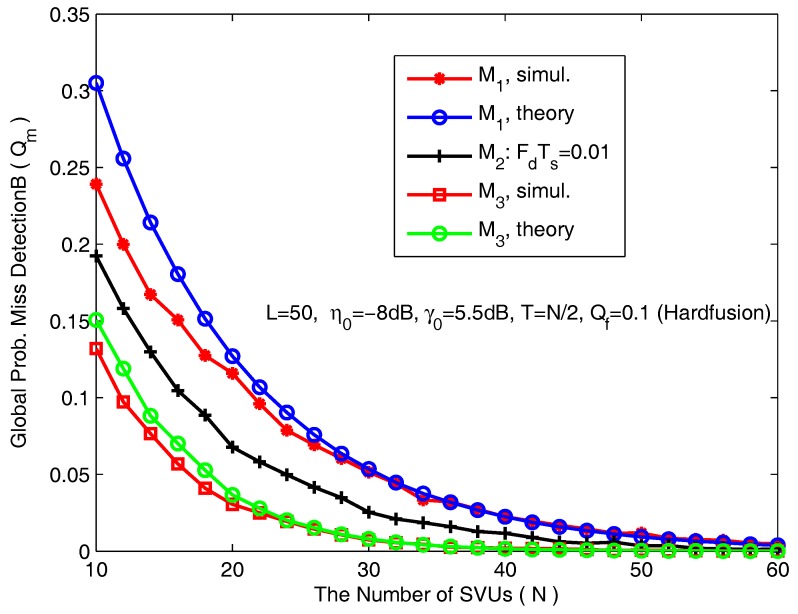
The global probability of misdetection as a function of the number of SVUs.

Secondly, we turn to cooperative scenarios. In Figure 6, we plot the global probability of misdetection ($Q_{m}$) as a function of the number of SVUs for hard fusion based on counting rule. The simulation parameters are *L* = 50, $\eta_{0}$ = −8 dB, $\gamma_{0}$ = 5.5 dB, T=N/2 and $Q_{f}$ = 0.1. As expected, the global misdetection probability decreases along with the increase of the number of SVUs. When *N* is extremely big, the global mis-detection probability approaches 0. For hard fusion, Figure 6 clearly demonstrates that the derived probability of misdetection based on Lindberg-Feller approximation is relatively poorer for a smaller number of SVUs. However, along with an increasing number of SVUs, the approximation asymptotically grows closer and closer to the exact sensing performance.

**Figure 7:**
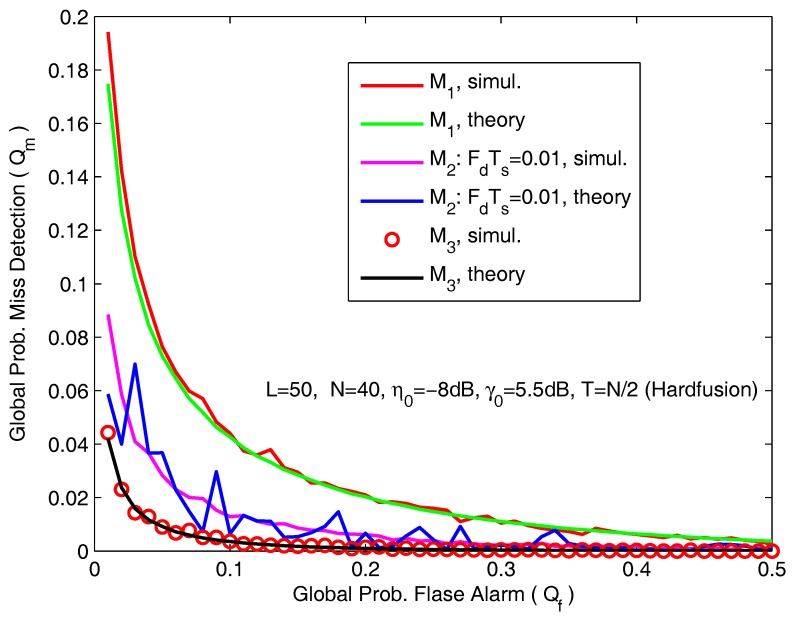
ROC curves for different channel model.

In Figure 7, we present the ROC curves for different channel models. The simulation parameters are *L* = 50, *N* = 40, $\eta_{0}$ = −8 dB and T=N/2. Note that, in the case of $M_{1}$ and $M_{3}$, we obtain the average misdetection probability for local sensing, allowing for a much smoother global probability curve than the case of $M_{2}$.

**Figure 8:**
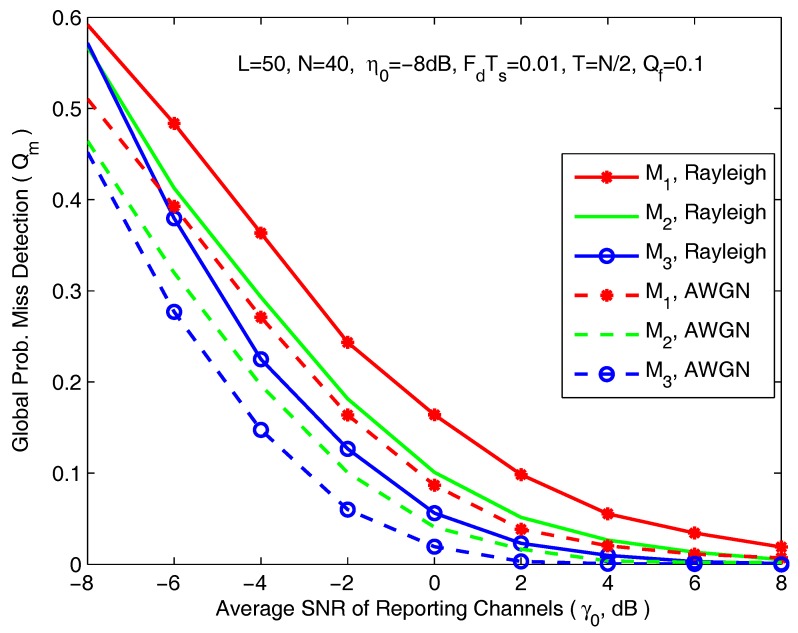
The global probability of misdetection as a function of average SNR of reporting channels.

In Figure 8, we present the average $Q_{m}$ curves as a function of the average SNR of reporting channels. The simulation parameters are *L* = 50, *N* = 40, $\eta_{0}$ = −8 dB, $F_{d}T_{s}$ = 0.01, T=N/2 and $Q_{f}$ = 0.1. In the condition of poor reporting SNR, large bit error occurs, which severely degrades the detection performance. We can clearly see that cooperative sensing performance improves quickly with the increase of the average SNR of reporting channel from −8 dB to 8 dB. Note that the sensing performance in AWGN channel is always better than in Rayleigh channel.

**Figure 9:**
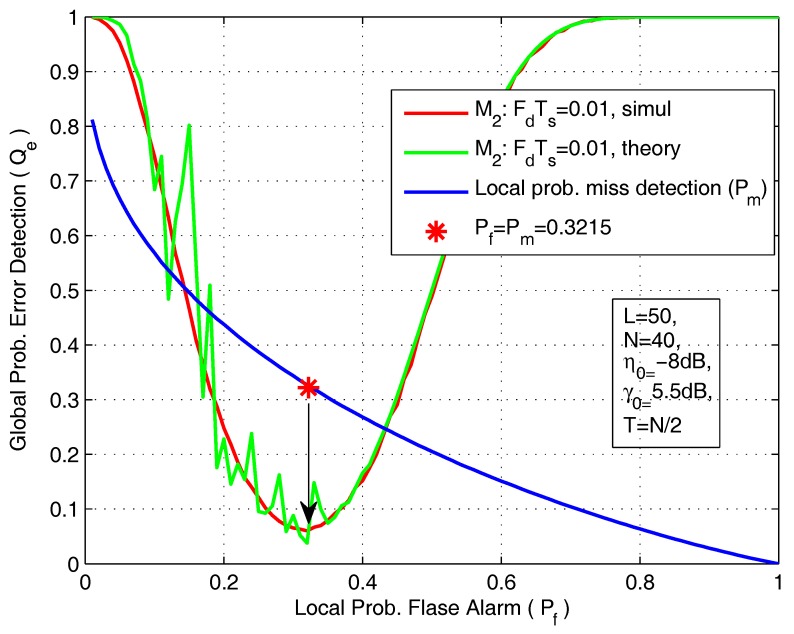
The global probability of error detection as a function of local probability of flase alarm.

**Figure 10:**
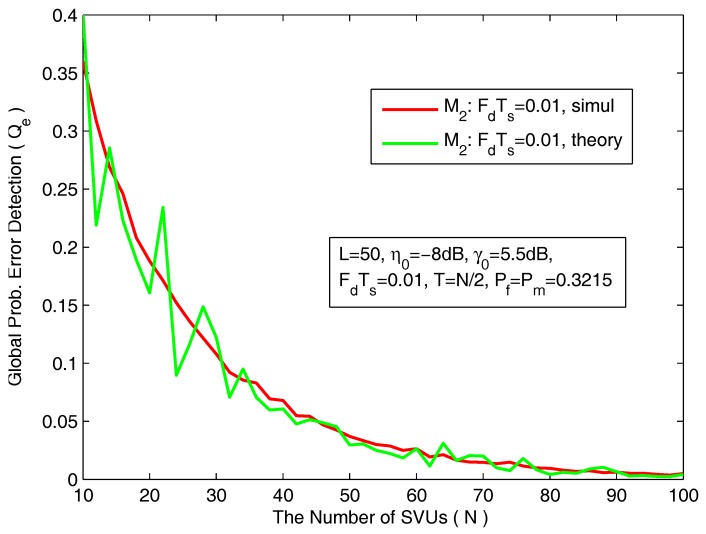
The global probability of error detection as a function of the number of SVUs.

In Figure 9, we plot the global probability of error detection ($Q_{e}$) as a function of the local false-alarm probability. The simulation parameters are *L* = 50, *N* = 40, $\eta_{0}$ = −8 dB, $\gamma_{0}$ = 5.5 dB, $F_{d}T_{s}$ = 0.01 and T=N/2. We can clearly see that, under certain conditions, there is an minimum probability of error detection, and that can be achieved when the local probabilities of false-alarm and mis-detection are equal ( $P_{f}=P_{m}$ ). In Figure 10, we plot the minimum achievable probability of error detection ($Q_{e}$) as a function of the number of SVUs.

## 6. Conclusions

In this paper, we have researched the application of cognitive radio technique to vehicular environments for the purpose of improving the reliability for vehicular communications. For this, we evaluated the detection performance of spectrum sensing in mobile vehicular environments. Simulation results demonstrate that temporal correlation cannot be neglected and be one of the considerable factors that may impact the sensing performance. In particular, high mobility of the vehicles provides the opportunity to exploit temporal diversity at each vehicle. In spite of the fact that cooperative sensing has normally been considered as a viable means of producing better detection performance by making the most of spatial diversity among vehicles, we do believe that due to high mobility, temporal diversity may be used in preference over vehicles’ cooperation in the future.

## Appendix A

In the following, we make clear how the two sufficient conditions (38) and (39) for the LF-CLT are satisfied for $\Lambda =\sum_{i=1}^{N}v_{i}$ under hypothesis $H_{j}$, for j=0,1. Notice that in the LF-CLT, essentially, the objective of the two conditions is to ensure that no single random variable is dominate in its contribution to the summation operator.

In the first step we shall prove that the first condition (38) is satisfied. As can be seen from (21) and (34), the variance of $v_{i}$, for i=1,2,⋯,N, under the hypotheses $H_{1}$, can be represented as

(A1) $$\begin{array}{cc} D[v_{i}\left|H_{1}\right.] & =P_{Di}\left(1-P_{Di}\right)\\ & =\left[\frac{1}{2}+\left(P_{di}-\frac{1}{2}\right)\sqrt{\frac{\gamma_{i}}{1+\gamma_{i}}}\right]\left[\frac{1}{2}-\left(P_{di}-\frac{1}{2}\right)\sqrt{\frac{\gamma_{i}}{1+\gamma_{i}}}\right]\\ & =\frac{1}{4}-{\left(P_{di}-\frac{1}{2}\right)}^{2}\left(\frac{\gamma_{i}}{1+\gamma_{i}}\right). \end{array}$$

It is sufficient to show that, under the hypothesis $H_{1}$, the variance of $v_{i}$ is lower bounded by a positive value, as long as the $P_{di}$ are bounded away from 0 and 1. Certainly, the $P_{di}$ can’t be equal to 0 and 1 in the practical vehicular networks.

In the next step, we show that the second condition (39) is satisfied. Let us make use of the fact that $v_{i}$ is a binary random variable which follows a Bernoulli distribution characterized by the associated $P_{Fi}$ and $P_{Di}$. It is plain enough that under the hypotheses $H_{1}$,

(A2) $$\begin{array}{cc} E\left[|v_{i}-E\left[v_{i}\right]{|}^{3}\left|H_{1}\right.\right] & =P_{Di}\left(1-P_{Di}\right)\left(P_{Di}^{2}+{\left(1-P_{Di}\right)}^{2}\right)\\ & <P_{Di}\left(1-P_{Di}\right). \end{array}$$

A similar analysis can be conducted, $P_{Di}\left(1-P_{Di}\right)$ is upper bounded by a positive value.

In the above analysis, we prove that the two sufficient conditions (38) and (39) in the LF-CLT are satisfied under the assumption of hypothesis $H_{1}$ explicitly, but the same proved method can be carried out to demonstrate the effectiveness under the hypothesis $H_{0}$.
